# A Combined Long Noncoding RNA Signature as a Candidate Prognostic Biomarker for Ovarian Cancer

**DOI:** 10.3389/fonc.2021.624240

**Published:** 2021-05-27

**Authors:** Hui Li, Shuoer Wang, Qianlan Yao, Yan Liu, Jing Yang, Lun Xu, Gong Yang

**Affiliations:** ^1^ Central Laboratory, the Fifth People's Hospital of Shanghai, Fudan University, Shanghai, China; ^2^ Department of Musculoskeletal Oncology, Fudan University Shanghai Cancer Center, Shanghai, China; ^3^ Department of Nuclear Medicine, Fudan University Shanghai Cancer Center, Shanghai, China; ^4^ Department of Oncology, Shanghai Medical College, Fudan University, Shanghai, China; ^5^ Department of Pathology, Fudan University Shanghai Cancer Center, Shanghai, China; ^6^ Institute of Pathology, Fudan University, Shanghai, China; ^7^ Department of Gynecology, Weifang People’s Hospital, Weifang, China; ^8^ Department of Anesthesiology and Postanesthesia Care Unit, the First Affiliated Hospital, Zhejiang University School of Medicine, Hangzhou, China; ^9^ Cancer Institute, Fudan University Shanghai Cancer Center, Shanghai, China

**Keywords:** long non-coding RNA, prognostic biomarker, ovarian cancer, bioinformatics analysis, TCGA

## Abstract

**Aims:**

Dysregulated long noncoding RNAs (lncRNAs) contributing to ovarian cancer (OC) development may serve as prognostic biomarker. We aimed to explore a lncRNA signature to serve as prognostic biomarker of OC.

**Methods:**

Univariate Cox regression was conducted on the lncRNA expression dataset from the TCGA cohort, and 246 genes significantly associated with survival were retained for building a model. A random forest survival model was carried out, and a model was developed using 6 genes with the highest frequency. The selected genes were applied in a Cox multivariate regression model for prognostic prediction by calculating the risk score. We also used CCK-8, EdU, and colony formation assays to validate the function of these lncRNAs in OC cells.

**Results:**

This study confirmed that the 6-lncRNA combined signature was related to OC prognosis. Systematic analysis demonstrated that lncRNA-associated genes were enriched in oncogenic signalling pathways. Five out of the 6 lncRNAs participated in OC proliferation.

**Conclusion:**

We established a 6-lncRNA combined signature for OC prognosis, which may serve as powerful prognostic biomarker for OC after further validation.

## Introduction

Ovarian cancer (OC) is a major cause of gynaecologic cancer death in women worldwide (1). Each year, 238,700 patients are newly diagnosed with OC, and 151,190 patients die of OC worldwide (2). The overall mortality of OC remains high owing to the high recurrence rate and lack of early detection (3). Thus, it is crucial to explore more reliable prognostic factors for early detection.

Long noncoding RNAs (lncRNAs) are a class of noncoding RNAs with a length of more than 200 nucleotides. Several lncRNAs have been confirmed to be deregulated and act as critical regulators in OC (4). Overexpression of lncRNA HOTTIP promoted the growth and metastasis of OC via the miR-615-3p/SMARCE1 axis (5). LncRNA HOTAIR maintained higher OC stemness through the miR-206/TBX3 pathway (6). Recently, several lncRNAs were discovered as prognostic biomarkers for OC patients. LncRNA PTPRG-AS1 overexpression could predict a poor prognosis of OC patients (7). The enhanced level of lncRNA ROR was positively correlated with poor clinical outcome of OC patients (8). However, these single features appear to have poor generality in new datasets (9). In this work, we identified a multiple lncRNA combined signature based on a prognostic model for OC patients and investigated the role of the signature on OC proliferation.

## Methods

### Cell Lines and Reagents

The human OC cell lines Hey and SKOV3 and the lentiviral packaging cell line 293T were obtained from the American Type Culture Collection (ATCC). RPMI 1640 medium (Sigma-Aldrich) was used to incubate Hey and SKOV3 cell lines, and DMEM was used to culture 293T cells. Both media were supplemented with 10% foetal bovine serum (FBS) and 1% penicillin/streptomycin. All cells were incubated in a humidified atmosphere at 37°C with 5% CO_2_.

### Plasmid Construction and Lentivirus Infection

According to the manufacturer’s protocol, two small hairpin RNAs (shRNAs) targeting each lncRNA were inserted into the lentiviral vector pLKO.1-puro to silence lncRNA expression. **Supplementary Table 1** shows the shRNA sequences used in this study. We used pLKO.1-puro scrambled shRNA as a control. In the presence of polybrene, Hey and SKOV3 cell lines were infected with the virus. Then, the medium containing puromycin was used to select Hey and SKOV3 stable clones. RT-PCR was used to confirm the knockdown efficiency of these shRNAs in Hey and SKOV3 cell lines.

### Reverse Transcription and Quantitative Real-Time PCR

TRIzol reagent (Invitrogen) was used to extract total RNA according to the manufacturer’s instructions. A NanoDrop 2000 (Thermo Fisher Scientific) was applied to measure the quantity and purity of the total RNA. The PrimeScript RT Master Mix kit (TAKARA) was used to synthesize cDNA. Real-time PCR was performed using FastStart Universal SYBR Green Master Mix (Rox) (Roche) in an ABI PRISM 7900 sequence detector (Applied Biosystems, Carlsbad, CA), and GAPDH was applied as an internal control. Relative mRNA levels were computed according to the Ct values relative to GAPDH. **Supplementary Table 2** shows the primers used in this study.

### Cellular Growth Assay

In total, 1000 SKOV3 cells or 1000 Hey cells were seeded in 96-well plates. Cell growth was examined by Cell Counting Kit-8 (CCK-8) (Beyotime, China). Briefly, the culture medium was removed at 24, 48, 96, and 120 h, and 10 μl of CCK-8 in 100 μl of medium was added to each well. Then, the cells were incubated at 37°C for 2 h, and the absorbance was measured at 450 nm. These experiments were repeated 3 times.

### Colony Formation Assay

A total of 800 SKOV3 cells or 500 Hey cells were seeded in 6-well plates for plate colony formation. Each cell type was cultured at 37°C in a 5% CO2 atmosphere for 2 weeks. Then, 4% PFA (paraformaldehyde) was used to fix the colonies for 10 min, and the colonies were stained with crystal violet solution for 20 min. After washing three times with PBS, the number of colonies was counted. The assays were repeated 3 times.

### Edu Cell Proliferation Assay

SKOV3 cells or Hey cells were seeded in 12-well plates. Then, the cells were cultured 24 h before EdU (C0078S, Beyotime, Shanghai, China) treatment. EdU was added in medium and incubated at 37°C for 2 h. PFA (4%) was used to fix the cells for 20 minutes, and 0.3% Triton X-100 was used to permeabilize the cells for 15 minutes. The cells were then incubated with Click reaction buffer for EdU staining for 30 min. Hoechst 33342 (5 μg/mL) was used to stain the cells at 37°C for 10 minutes, followed by PBS washes 3 times. Images were captured using a fluorescence microscope.

### Dataset Acquisition and Preprocessing

The TCGA lncRNA dataset was downloaded from the TANRIC database (https://bioinformatics.mdanderson.org/public-software/tanric/). Clinical information and mRNA expression datasets of the corresponding patients were retrieved from UCSC Xena (http://xena.ucsc.edu/public-hubs/). The lncRNA and mRNA expression datasets were first log2 scaled and then z-score transformed for further analysis.

### Gene Selection and Model Development

The lncRNA expression dataset of the TCGA cohort was analysed by univariate Cox regression. Model building used 246 genes significantly related to survival (*P* < 0.05). A random forest survival model was carried out with parameters of 100 iterations and 1000 trees per iteration to optimize the panel. The model development used six genes with the highest frequency. The selected genes were implemented in a Cox multivariate regression model. The risk score was calculated using the following formula:

$$\begin{array}{c} \text{Risk}\;\text{score}=(1.3546315*\text{AL}121820.1)+(14.5598208*\text{linc}01984-201)\\ +(0.7082245*\text{AC}006262.3)+(-16.8262818*\text{linc}02115)\\ +(0.1061679*\text{AL}713998.1)+(-2.2946211*\text{AL}138831.2). \end{array}$$

### Statistical Analysis

R (version 4.0.2) was used to analyse the data in this study. The R package “survival” was implemented in univariate Cox regression, survival analysis, and multivariate regression. The R package “randomForestSRC” was applied to the random forest survival model and risk score. The AUC and ROC were calculated with the R package “ROCR”. Correlation analysis was calculated by Pearson correlation coefficients, and gene enrichment was implemented using Metascape (http://metascape.org/gp/index.html). A two-sided *P* value < 0.05 was considered significant in the study.

## Results

### Feature Selection and Prognostic Value of lncRNAs in the TCGA Cohort

**Figure 1:**
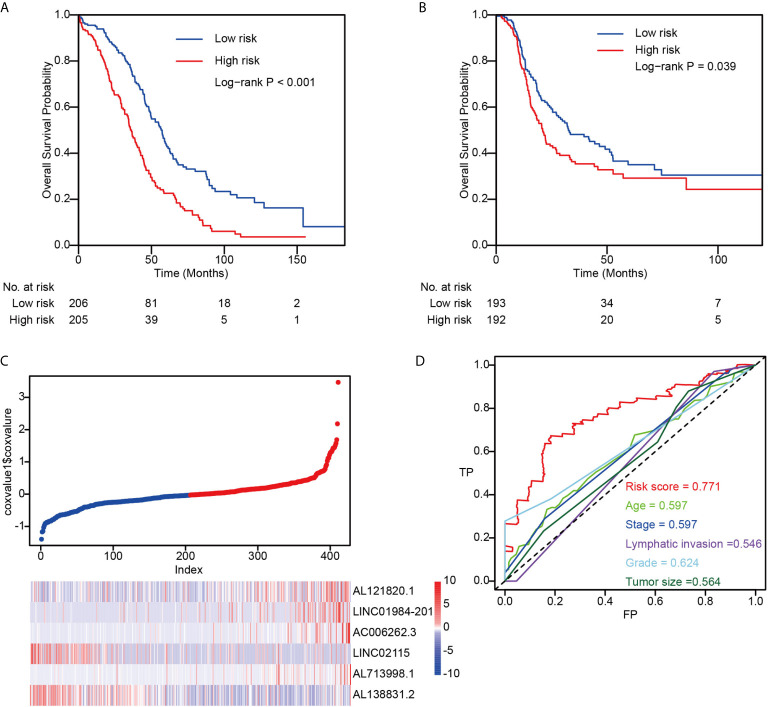
The performance of risk score in TCGA dataset. **(A)** The overall survival difference between low-risk and high-risk groups; **(B)** The progression-free survival difference between low-risk and high-risk groups; **(C)** Detailed survival information and gene expression patterns in TCGA dataset; **(D)** Three years survival ROC by using risk score, age, stage, lymphatic invasion, grade, and tumor size.

To identify lncRNAs related to OC prognosis, we first performed univariate Cox regression on the TCGA dataset (n = 411). Genes significantly related to overall survival were reserved for further analysis, and a random survival forest model was built on these genes for prognosis prediction (Methods). Finally, the six genes with the highest prediction accuracy were selected for the optimization of the model, and a multivariate Cox proportional hazard model was constructed to calculate the risk score for each sample (Method). The patients were separated into low-risk and high-risk groups by the median split of risk scores, and the high-risk group demonstrated significantly worse overall survival probability (**Figure 1A**) and progression-free survival probability (**Figure 1B**). Interestingly, we found that four lncRNAs were positively correlated with high-risk scores (AL121820.1, AC006262.3, LINC02115 and AL138831.2), whereas the other two lncRNAs were negatively correlated with the risk score (LINC01984-201 and AL713998.1) (**Figure 1C**). Compared with clinical factors, including age, tumour stage, lymphatic invasion, grade, and tumour size, the risk score showed much higher area under the curve (AUC) values in the prognostic prediction of OC patients (**Figure 1D**), indicating the possibility of the risk score as a new prognostic biomarker in clinical application.

### Targeted Genes of lncRNAs Constructed the Risk Score

**Figure 2:**
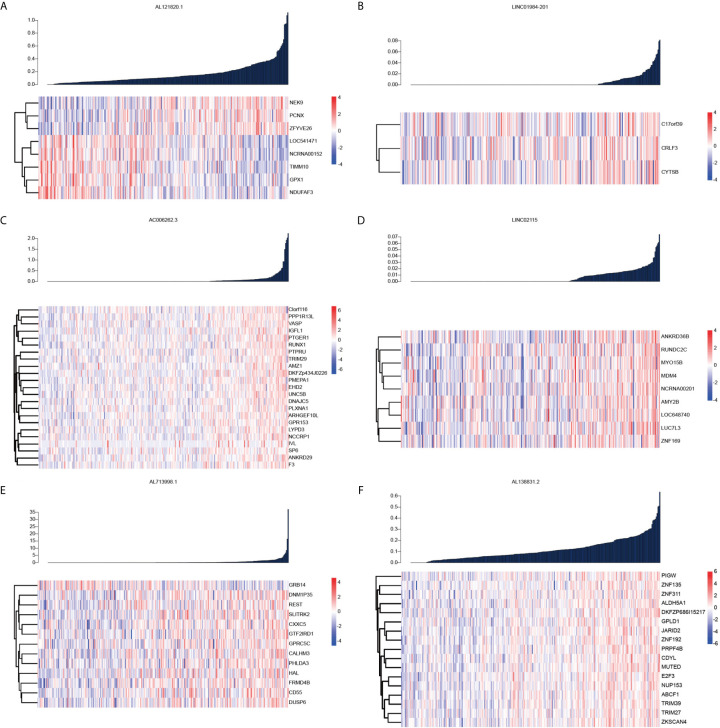
Pearson correlation coefficient analysis between the expression levels of coding genes and the six lncRNAs. **(A)** Heatmap of the coding genes expression targeted by AL121820.1; **(B)** Heatmap of the coding genes expression targeted by linc01984-201; **(C)** Heatmap of the coding genes expression targeted by AC006262.3; **(D)** Heatmap of the coding genes expression targeted by linc02115; **(E)** Heatmap of the coding genes expression targeted by AL713998.1; **(F)** Heatmap of the coding genes expression targeted by AL138831.2.

To identify genes targeted by the six lncRNAs showing prognostic value in OC, we next conducted Pearson correlation coefficient analysis between the expression levels of coding genes and the six lncRNAs. The results showed that NEK9, PCNX, and ZFYVE26 were positively correlated with AL121820.1, whereas TIMM10, GPX1, and NDUFAF3 were negatively associated with the expression of AL121820.1, demonstrating the biphasic regulation of AL121820.1 to the targeted genes (**Figure 2A**). In contrast, genes showing high correlation coefficients (R > 0.4) with the other five lncRNAs were most positively correlated with their corresponding lncRNAs (**Figures 2B–F**). Specifically, CRLF3, which encodes a cytokine receptor-like factor that may negatively regulate cell cycle progression, was positively correlated with LINC01984-201 (10) (**Figure 2B**). Twenty-three genes demonstrated a positive correlation with AC006262.3 (**Figure 2C**), and MYO15B and ZNF169 were positively correlated with LINC02115 (**Figure 2D**). Except for GRB14, which was associated with the immune response IL-23 signalling pathway, and cell surface interactions in the vascular wall, the other 13 genes were positively correlated with AL713998.1 (**Figure 2E**). Zinc finger proteins ZNF135 and ZNF311 were both positively correlated with AL138831.2 (**Figure 2F**).

**Figure 3:**
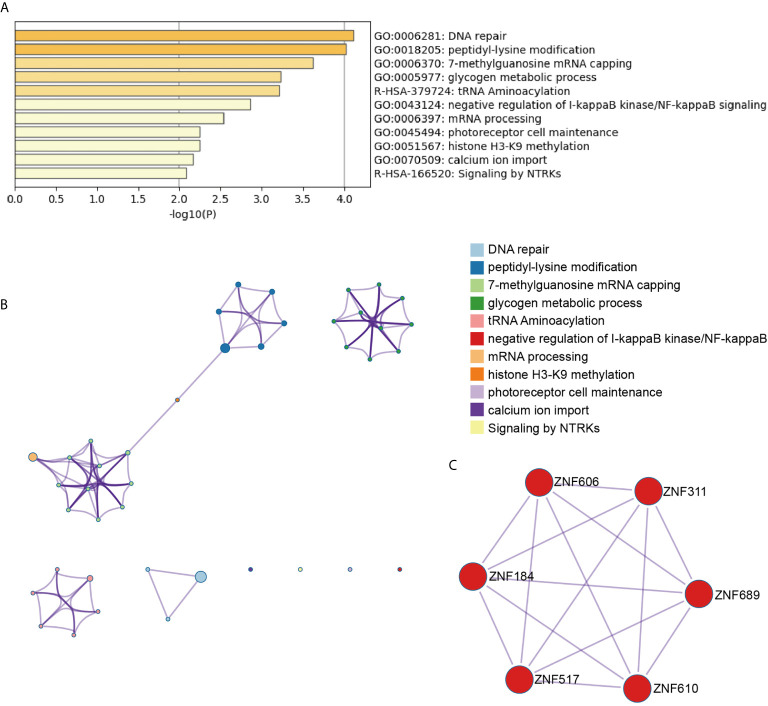
Gene enrichment analysis of the AL138831.2-signature. **(A)** The histogram of the top 11 enriched pathways associated with risk score in AL138831.2 was arranged by -Log10P value. Each bar represented one enriched term and was coloured by -Log10P value; **(B)** Using Metascape to cluster the pathways related with AL138831.2. The top 11 enriched pathways were shown (right panel); **(C)** The pathways related with AL138831.2 were clustered by MCODE ZNF complexes.

**Figure 4:**
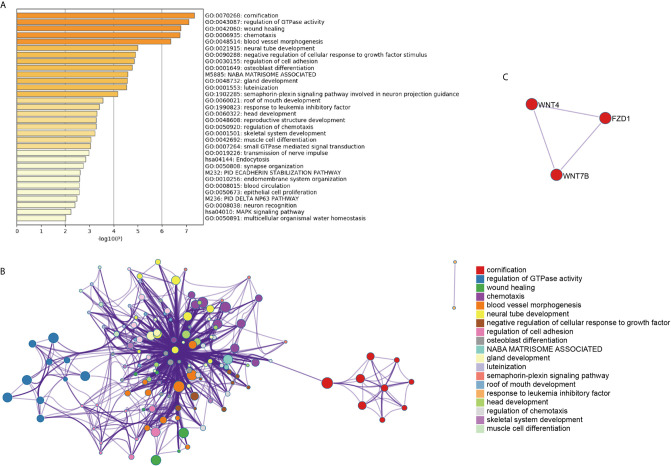
Gene enrichment analysis of the AC006262.3-signature. **(A)** The histogram of the top 19 enriched pathways associated with risk score in AC006262.3 was arranged by -Log10P value. Each bar represented one enriched term and was coloured by -Log10P value; **(B)** Using Metascape to cluster the pathways related with AC006262.3. The top 19 enriched pathways were shown (right panel); **(C)** The pathways related with AC006262.3 were clustered by MCODE ZNF complexes.

To systematically explore the roles of the lncRNA-associated genes, we further performed functional enrichment analysis on their mRNAs to conduct the significant correlation (*P* < 0.05) with the corresponding lncRNAs by Metascape (11). The AL138831.2-correlated genes were significantly enriched in DNA repair, peptidyl-lysine modification, and negative regulation of I-κB kinase/NF-κB signalling pathways (**Figures 3A, B**). In addition, the MCODE ZNF complexes were also enriched (**Figure 3C**), suggesting that AL138831.2 may interact with ZNF proteins in OV. Considering that the high expression of AL138831.2 was associated with poor prognosis in OV, AL138831.2 might also contribute to OC development through these oncogenic signalling pathways. The AC006262.3-associated genes were significantly enriched in the regulation of cellular response to growth factor stimuli, the regulation of cell adhesion, and the responses to leukaemia inhibitory factor and MAPK signalling pathway (**Figures 4A–C**).

### LncRNAs Reflected the Cell Motility Status

**Figure 5:**
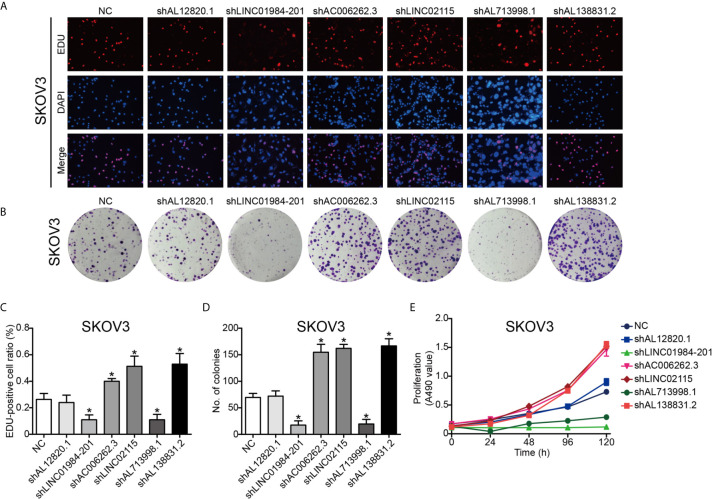
LncRNAs regulated the proliferation ability of SKOV3 cancer cell line. After knocking down of the LncRNAs, the proliferation ability of SKOV3 cancer cells was detected by Edu staining, colony formation and CCK8 assay. **(A)** Representative images of EDU staining; **(B)** Representative images of colony formation; **(C)** The quantification result of EDU staining; **(D)** The quantification result of colony formation; **(E)** The cell viability inspected by CCK8 assay. P^*^<0.05 *vs*. NC. Data were from three independent experiments.

**Figure 6:**
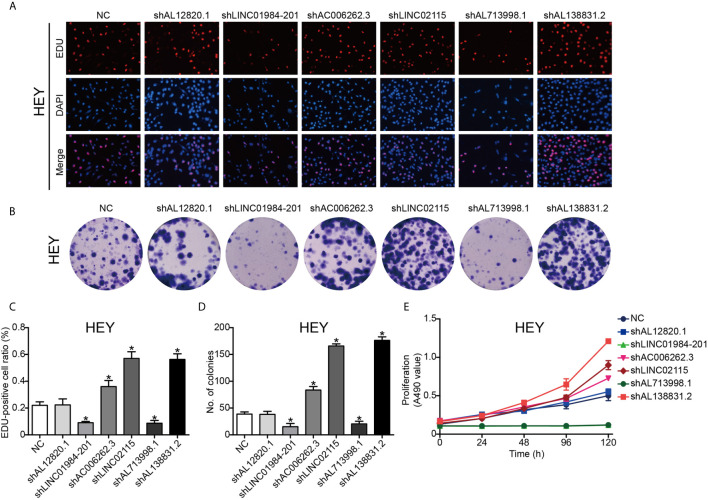
LncRNAs regulated the proliferation ability of HEY cancer cell line. After knocking down of the LncRNAs, the proliferation ability of HEY cancer cells was detected by Edu staining, colony formation and CCK8 assay. **(A)** Representative images of EDU staining; **(B)** Representative images of colony formation; **(C)** The quantification result of EDU staining; **(D)** The quantification result of colony formation; **(E)** The cell viability inspected by CCK8 assay. P^*^<0.05 *vs*. NC. Data were from three independent experiments.

To validate the function of the six identified lncRNAs in OC development, we silenced the expression of each of these lncRNAs in the OC Hey and SKOV3 cell lines by shRNAs. The expression of lncRNAs detected by q-PCR was remarkably decreased in OC cells (**Supplementary Figure 1**). The EdU assay was used to study the effects of lncRNAs on cell proliferation. The results from EdU assays showed that silencing AC006262.3, LINC02115, and AL138831.2 indeed promoted the proliferation of Hey and SKOV3 cells in comparison with that of the control cells (**Figures 5A, C** and **6A, C**). Consistent with the proliferation assay, silencing of AC006262.3, LINC02115, and AL138831.2 increased the colony number, and silencing of LINC01984-201 and AL713998 decreased the colony number in both Hey and SKOV3 cells compared with that of the control cells (**Figures 5B, D** and **6B, D**). As shown in **Figures 5E** and **6E**, the CCK-8 assay indicated that silencing AC006262.3, LINC02115, and AL138831.2 in Hey and SKOV3 cells promoted cell growth, but knockdown of LINC01984-201 and AL713998 restrained cell growth compared with that of the control cells. However, the role of AL12820.1 in cell proliferation was not obvious.

## Discussion

This study carried out a multistep analysis of a lncRNA signature in OC. Based on the lncRNA expression dataset of the TCGA cohort, we used a univariate Cox regression and a random forest survival model to establish a prognostic 6-lncRNA signature in OC. Functional analysis showed that tumour-related processes were significantly enriched. In addition, CCK-8, EdU proliferation and colony formation assays were performed to validate the function of these lncRNAs. Thus, the 6-lncRNA combined signature is a robust model that could serve as a novel biomarker for OC prognosis.

As a common gynaecologic malignancy, OC is the leading cause of cancer death in women worldwide (12). OC is a histologically heterogeneous cancer caused by variations in genetic and environmental factors (13). An increasing body of evidence suggests that dysregulated lncRNAs play an important role in the progression of OC (14). As shown in **Figure 2**, lncRNAs could regulate the coding genes expression in OC. The mechanisms by which lncRNAs regulate the expression of the selected mRNAs may include RNA stability regulation (15), mRNA translation (16), chromatin modification (17), transcription activation (17, 18) and transcription interference (19). The abnormal expression of lncRNAs could promote the proliferation, migration, invasion, and metastasis of OC by regulating the mRNAs expression and contribute to poor prognosis of OC (20). It has been confirmed that the AC006262.3 related genes such as VASP (21), RUNX1 (22), TRIM29 (23) in **Figure 2C** contribute to the progression of OC. Thus, the aberrant expression of lncRNAs may serve as new prognostic biomarkers in OC.

With the rapid development of high-throughput technologies such as gene chip and RNA sequencing, gene analysis has become a powerful tool for screening molecular biomarkers of tumour prognosis prediction (24). Recently, several studies demonstrated that the robustness of several biomarker combinations is better than the robustness of a single biomarker (25). Our study analysed the available RNA‐seq data of OC from TCGA database and established a robust 6‐lncRNA signature that could serve as prognostic factor. As shown in **Figure 3**, the AL138831.2-related gene were indeed enriched in tumour-related networks, including “DNA repair”, “negative regulation of NF-κB signalling”, “glycogen metabolic process” and “ZNF proteins”, indicating that the activation of these pathways may promote higher mortality risk in patients with high-risk scores. ZNF proteins are immensely implicated in the development of several tumours including OC (26). Through the ZNF proteins, AL138831.2 might function in OC cell metastasis and serve as a prognosis-associated biomarker in patients with OC. Moreover, Metascape and MCODE ZNF complexes analyses in **Figure 4** showed that the mRNAs which are positively co-expressed with AC006262.3 participate in “would healing”, “cell adhesion” and “WNT signalling pathway”, which are considered to be correlated with OC metastasis. Aberrant activation and upregulation of WNT signalling pathway could contribute to the progression of OC and lead to poor prognosis of OC (27). This indicated that AC006262.3 might function in the prognosis of patients with OC by regulating WNT signalling pathway. These results reconfirmed that lncRNAs could act as new biomarkers of OC and provide evidence for the clinical application of these 6 lncRNAs. Furthermore, a recent study analysed the role of lncRNAs in BRCA mutant ovarian cancer (28) and then we searched BRCA mutation information in 294 samples of our study. We found that (1) BRCA mutation rates was very low (**Supplementary Figure 2A**); (2) BRCA mutation did not affect the overall survival probability of ovarian cancer patients (P=0.506, **Supplementary Figure 2B**). BRCA mutation had no effect on integration results. Therefore, BRCA mutation has no relationship with LncRNAs screened by this study.

Since the risk score was calculated based on the expression of candidate genes, the effectiveness of the model relies on the function of these genes. However, there have been no studies to report the biological functions of these 6 lncRNAs at present. Thus, we preliminarily explored the biological functions of these 6 lncRNAs. As shown in **Figures 5** and **6**, we found that silencing AC006262.3, LINC02115, and AL138831.2 promoted cell growth and that knockdown of LINC01984-201 and AL713998 retained cell growth. However, the role of AL12820.1 in cell proliferation was not clear. Collectively, these results indicate that the genes selected in the model are functional active for proliferation, and thus predict survival in OC.

There are some limitations in our study. (1) Our data were not eligible to show the pathophysiological functions of these lncRNAs in our study. Thus, how these lncRNAs take part in cancer development may need to be validated in the future. (2) We are unclear why AL12820.1 did not affect the proliferation of OC cells. (3) The underlying mechanisms of these lncRNAs in OC need to be further investigated.

## Conclusion

In conclusion, our study identified a 6-lncRNA combined signature associated with the prognosis of OC. Systematic analysis found that lncRNA-associated genes were enriched in oncogenic signalling pathways. Furthermore, five out of six lncRNAs were found to regulate the proliferation of OC cells.

## Data Availability Statement

The datasets presented in this study can be found in online repositories. The names of the repository/repositories and accession number(s) can be found in the article/**Supplementary Material**.

## Ethics Statement

Ethical approval has been obtained from the Fudan University Shanghai Cancer Center Ethics Committee.

## Author Contributions

HL, SW and QY mainly took charge of researching, organizing data and writing the manuscript; YL polished the language in the review process; JY and LX used illustration software to arrange data; GY designed this study. All authors contributed to the article and approved the submitted version.

## Funding

This study was supported by grants from the National Natural Science Foundation of China (81372797 for GY).

## Conflict of Interest

The authors declare that the research was conducted in the absence of any commercial or financial relationships that could be construed as a potential conflict of interest.

## Supplementary Material

Supplementary Figure 1 The knocking down efficiency of shRNAs for these 6 lncRNAs in SKOV3 and HEY cancer cell lines. **(A)**The knocking down efficiency of shRNAs for these 6 lncRNAs in SKOV3 cancer cell; **(B)** The knocking down efficiency of shRNAs for these 6 lncRNAs in HEY cancer cell.

Supplementary Figure 2 Information about somatic BRCA mutant status of tumor cells. **(A)** BRCA mutant status in 294 samples of ovarian cancer; **(B)** The overall survival difference between low-risk and high-risk groups.

## References

[1] Torre LA Trabert B DeSantis CE Miller KD Samimi G Runowicz CD. Ovarian Cancer Statistics, 2018. CA Cancer J Clin (2018) 68:284–96. 10.3322/caac.21456 PMC6621554 29809280

[2] Chornokur G Amankwah EK Schildkraut JM Phelan CM. Global Ovarian Cancer Health Disparities. Gynecol Oncol (2013) 129:258–64. 10.1016/j.ygyno.2012.12.016 PMC3608795 23266352

[3] Jochum F Vermel M Faller E Boisrame T Lecointre L Akladios C. Three and Five-Year Mortality in Ovarian Cancer After Minimally Invasive Compared to Open Surgery: A Systematic Review and Meta-Analysis. J Clin Med (2020) 9:2507. 10.3390/jcm9082507 PMC7463583 32759715

[4] Si L Chen J Yang S Liu Z Chen Y Peng M. lncRNA HEIH Accelerates Cell Proliferation and Inhibits Cell Senescence by Targeting miR-3619-5p/CTTNBP2 Axis in Ovarian Cancer. Menopause (2020) 27:1302–14. 10.1097/GME.0000000000001655 33110047

[5] Wu H Wei HY Chen QQ. Long Noncoding RNA HOTTIP Promotes the Metastatic Potential of Ovarian Cancer Through the Regulation of the miR-615-3p/SMARCE1 Pathway. Kaohsiung J Med Sci (2020) 36:973–82. 10.1002/kjm2.12282 PMC11896243 32783402

[6] Zhang Y Guo J Cai E Cai J Wen Y Lu S. HOTAIR Maintains the Stemness of Ovarian Cancer Stem Cells Via the miR-206/TBX3 Axis. Exp Cell Res (2020) 395:112218. 10.1016/j.yexcr.2020.112218. 32771526

[7] Ren XY Yang WB Tian Y. Overexpression of Long Noncoding RNA Ptprg-AS1 Is Associated With Poor Prognosis in Epithelial Ovarian Cancer. Rev Assoc Med Bras (1992) (2020) 66:948–53. 10.1590/1806-9282.66.7.948 32844927

[8] Shen W Xie X Liu M Wang L. Diagnostic Value of lncRNA ROR in Differentiating Ovarian Cancer Patients. Clin Lab (2020) 66:1261–7. 10.7754/Clin.Lab.2019.191035 32658434

[9] Liu G Chen L Ren H Liu F Dong C Wu A. Seven Genes Based Novel Signature Predicts Clinical Outcome and Platinum Sensitivity of High Grade IIIc Serous Ovarian Carcinoma. Int J Biol Sci (2018) 14:2012–22. 10.7150/ijbs.28249 PMC6299362 30585265

[10] Hahn N Büschgens L Schwedhelm-Domeyer N Bank S Geurten BRH Neugebauer P. The Orphan Cytokine Receptor CRLF3 Emerged With the Origin of the Nervous System and Is a Neuroprotective Erythropoietin Receptor in Locusts. Front Mol Neurosci (2019) 12:251. 10.3389/fnmol.2019.00251 31680856 PMC6797617

[11] Xie L Chao X Teng T Li Q Xie J. Identification of Potential Biomarkers and Related Transcription Factors in Peripheral Blood of Tuberculosis Patients. Int J Environ Res Public Health (2020) 17:6993. 10.3390/ijerph17196993 PMC7579196 32987825

[12] Behera A Ashraf R Srivastava AK Kumar S. Bioinformatics Analysis and Verification of Molecular Targets in Ovarian Cancer Stem-Like Cells. Heliyon (2020) 6:e04820. 10.1016/j.heliyon.2020.e04820 32984578 PMC7492822

[13] Leskela S Romero I Rosa-Rosa JM Caniego-Casas T Cristobal E Pérez-Mies B. Molecular Heterogeneity of Endometrioid Ovarian Carcinoma: An Analysis of 166 Cases Using the Endometrial Cancer Subrogate Molecular Classification. Am J Surg Pathol (2020) 44:982–90. 10.1097/PAS.0000000000001478 32384322

[14] Pal S Garg M Pandey AK. Deciphering the Mounting Complexity of the p53 Regulatory Network in Correlation to Long Non-Coding RNAs (lncRNAs) in Ovarian Cancer. Cells (2020) 9:527. 10.3390/cells9030527 PMC7140525 32106407

[15] He J Zuo Q Hu B Jin H Wang C Cheng Z. A Novel, Liver-Specific Long Noncoding RNA LINC01093 Suppresses HCC Progression by Interaction With IGF2BP1 to Facilitate Decay of GLI1 mRNA. Cancer Lett (2019) 450:98–109. 10.1016/j.canlet.2019.02.033 30790682

[16] Jia X Shi L Wang X Luo L Ling L Yin J. KLF5 Regulated Lncrna RP1 Promotes the Growth and Metastasis of Breast Cancer Via Repressing p27kip1 Translation. Cell Death Dis (2019) 10:373. 10.1038/s41419-019-1566-5 31073122 PMC6509113

[17] Xu M Xu X Pan B Chen X Lin K Zeng K. Lncrna SATB2-AS1 Inhibits Tumor Metastasis and Affects the Tumor Immune Cell Microenvironment in Colorectal Cancer by Regulating SATB2. Mol Cancer (2019) 18:135. 10.1186/s12943-019-1063-6 31492160 PMC6729021

[18] Zhang H Zhang N Liu Y Su P Liang Y Li Y. Epigenetic Regulation of NAMPT by NAMPT-AS Drives Metastatic Progression in Triple-Negative Breast Cancer. Cancer Res (2019) 79:3347–59. 10.1158/0008-5472.CAN-18-3418 30940661

[19] Liu M Zhong J Zeng Z Huang K Ye Z Deng S. Hypoxia-Induced Feedback of HIF-1α and lncRNA-CF129 Contributes to Pancreatic Cancer Progression Through Stabilization of p53 Protein. Theranostics (2019) 9:4795–810. 10.7150/thno.30988 PMC6643431 31367258

[20] Liu Y Xu B Liu M Qiao H Zhang S Qiu J. Long non-Coding RNA SNHG25 Promotes Epithelial Ovarian Cancer Progression by Up-Regulating COMP. J Cancer (2021) 12:1660–8. 10.7150/jca.47344 PMC7890321 33613753

[21] Nayak AP Pera T Deshpande DA Michael JV Liberato JR Pan S. Regulation of Ovarian Cancer G Protein-Coupled Receptor-1 Expression and Signaling. Am J Physiol Lung Cell Mol Physiol (2019) 316:L894–902. 10.1152/ajplung.00426.2018 PMC6589584 30724097

[22] Xiao L Peng Z Zhu A Xue R Lu R Mi J. Inhibition of RUNX1 Promotes Cisplatin-Induced Apoptosis in Ovarian Cancer Cells. Biochem Pharmacol (2020) 180:114116. 10.1016/j.bcp.2020.114116 32579960

[23] Hao L Wang JM Liu BQ Yan J Li C Jiang JY. M6a-YTHDF1-Mediated TRIM29 Upregulation Facilitates the Stem Cell-Like Phenotype of Cisplatin-Resistant Ovarian Cancer Cells. Biochim Biophys Acta Mol Cell Res (2021) 1868:118878. 10.1016/j.bbamcr.2020.118878 33011193

[24] Kumar S Patnaik S Dixit A. Predictive Models for Stage and Risk Classification in Head and Neck Squamous Cell Carcinoma (HNSCC). PeerJ (2020) 8:e9656. 10.7717/peerj.9656 33024622 PMC7518185

[25] Zhang Y Zhou H Zhang M Xing L Yang C Xia B. Integrated Analysis of a Competing Endogenous RNA Network Reveals an 11-lncRNA Prognostic Signature in Ovarian Cancer. Aging (Albany NY) (2020) 12(24):25153–71. 10.18632/aging.104116 PMC7803494 33223503

[26] Duan Z Choy E Harmon D Yang C Ryu K Schwab J. ZNF93 Increases Resistance to ET-743 (Trabectedin; Yondelis) and PM00104 (Zalypsis) in Human Cancer Cell Lines. PloS One (2009) 4:e6967. 10.1371/journal.pone.0006967 19742314 PMC2734182

[27] He S Wang W Wan Z Shen H Zhao Y You Z. FAM83B Inhibits Ovarian Cancer Cisplatin Resistance Through Inhibiting Wnt Pathway. Oncogenesis (2021) 10(1):6. 10.1038/s41389-020-00301-y 33423038 PMC7797002

[28] Pan Y Jia LP Liu Y Han Y Li Q Zou Q. A Novel Signature of Two Long non-Coding RNAs in BRCA Mutant Ovarian Cancer to Predict Prognosis and Efficiency of Chemotherapy. J Ovarian Res (2020) 13:112. 10.1186/s13048-020-00712-w 32950050 PMC7502206

