# Supplementary figures and images for: A Combined Long Noncoding RNA Signature as a Candidate Prognostic Biomarker for Ovarian Cancer
Source: Front Oncol. 2021 May 27;11:624240. doi: 10.3389/fonc.2021.624240 (PMC8191461; doi:10.3389/fonc.2021.624240)

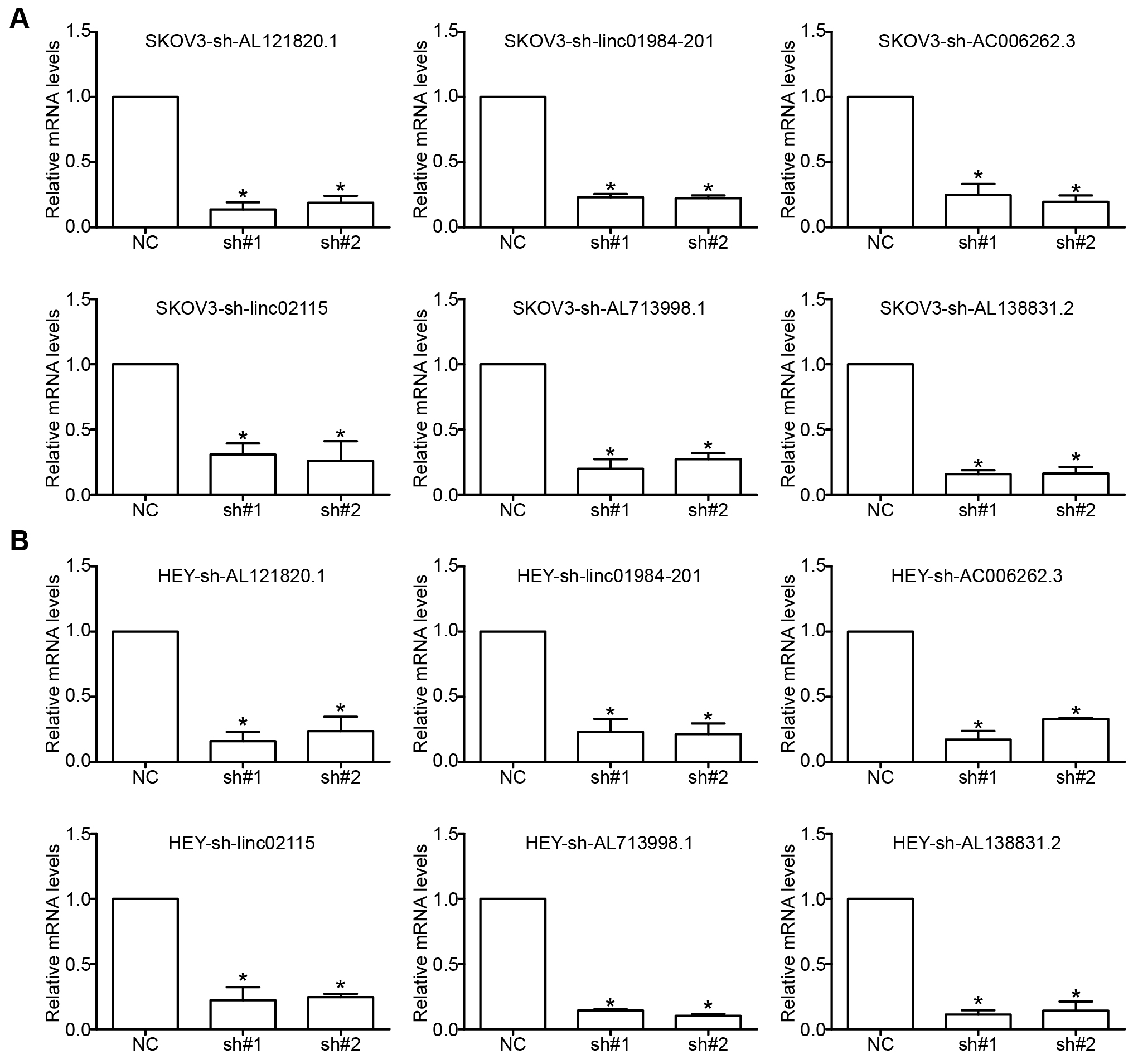

Supplement: Supplementary Figure 1 — The knocking down efficiency of shRNAs for these 6 lncRNAs in SKOV3 and HEY cancer cell lines. (A)The knocking down efficiency of shRNAs for these 6 lncRNAs in SKOV3 cancer cell; (B) The knocking down efficiency of shRNAs for these 6 lncRNAs in HEY cancer cell. [file Image_1.jpeg]

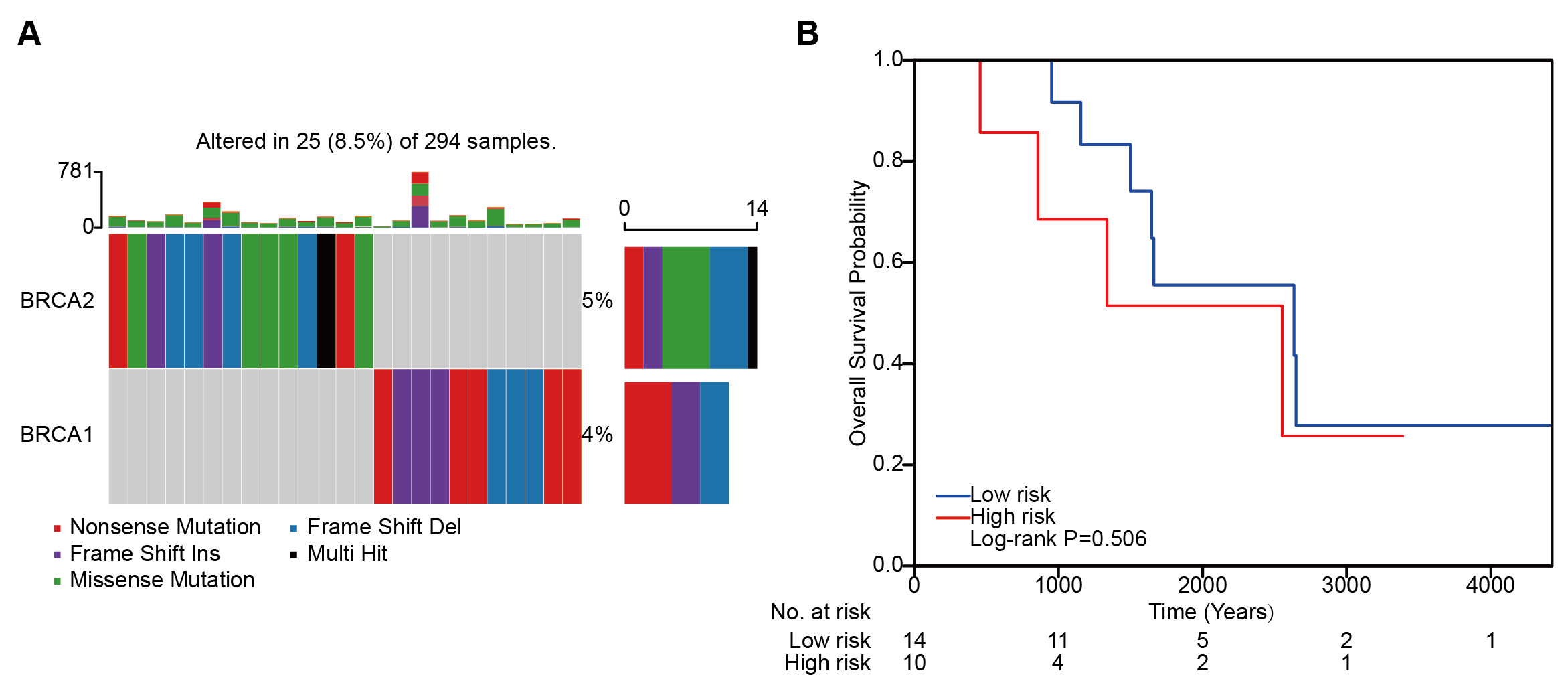

Supplement: Supplementary Figure 2 — Information about somatic BRCA mutant status of tumor cells. (A) BRCA mutant status in 294 samples of ovarian cancer; (B) The overall survival difference between low-risk and high-risk groups. [file Image_2.jpeg]
