# Supplementary material for: A Combined Long Noncoding RNA Signature as a Candidate Prognostic Biomarker for Ovarian Cancer
Source: Front Oncol. 2021 May 27;11:624240. doi: 10.3389/fonc.2021.624240 (PMC8191461; doi:10.3389/fonc.2021.624240)
Supplement: Supplementary file 3 [file DataSheet_1.pdf]

Table 1. Primers for the lncRNA shRNA in human ovarian cancer.

Gene	Sequence
LncAL12820.1-shRNA-1F	CCGGGCAATGCCTCACCCATTTAAACTCGAGTTTAAATGGGTGAGGCATTGCTTTTTG
LncAL12820.1-shRNA-1R	AATTCAAAAAGCAATGCCTCACCCATTTAAACTCGAGTTTAAATGGGTGAGGCATTGC
LncAL12820.1-shRNA-2F	CCGGGGGCTGTGTTGACAGTGATTTCTCGAGAAATCACTGTCAACACAGCCCTTTTTG
LncAL12820.1-shRNA-2R	AATTCAAAAAGGGCTGTGTTGACAGTGATTTCTCGAGAAATCACTGTCAACACAGCCC
Lnc01984-201-shRNA-1F	CCGGGCCATTATCCACTGAACTATTCTCGAGAATAGTTCAGTGGATAATGGCTTTTTG
Lnc01984-201-shRNA-1R	AATTCAAAAAGCCATTATCCACTGAACTATTCTCGAGAATAGTTCAGTGGATAATGGC
Lnc01984-201-shRNA-2F	CCGGGACTGTAGAAGAGAGGGTTAACTCGAGTTAACCCTCTCTTCTACAGTCTTTTTG
Lnc01984-201-shRNA-2R	AATTCAAAAAGACTGTAGAAGAGAGGGTTAACTCGAGTTAACCCTCTCTTCTACAGTC
LncAC006262.3-shRNA-1F	CCGGGATGCAGTGCTGAGTAGAACTCGAGTTCTACTCAGCACTGCATCTTTTTG
LncAC006262.3-shRNA-1R	AATTCAAAAAGATGCAGTGCTGAGTAGAACTCGAGTTCTACTCAGCACTGCATC
LncAC006262.3-shRNA-2F	CCGGGAGAGAGAAGGTAGGAGAACTCGAGTTCTCCTACCTTCTCTCTCTTTTTG
LncAC006262.3-shRNA-2R	AATTCAAAAAGAGAGAGAAGGTAGGAGAACTCGAGTTCTCCTACCTTCTCTCTC
Lnc02115-shRNA-1F	CCGGGAGTATGACTGCATCTTTACTCGAGTAAAGATGCAGTCATACTCTTTTTG
Lnc02115-shRNA-1R	AATTCAAAAAGAGTATGACTGCATCTTTACTCGAGTAAAGATGCAGTCATACTC
Lnc02115-shRNA-2F	CCGGGACAAGAGAGACCTTCCTACTCGAGTAGGAAGGTCTCTCTTGTCTTTTTG
Lnc02115-shRNA-2R	AATTCAAAAAGACAAGAGAGACCTTCCTACTCGAGTAGGAAGGTCTCTCTTGTC
LncAL713998.1-shRNA-1F	CCGGGCTCTGTAACCTTGTGATACTCGAGTATCACAAGGTTACAGAGCTTTTTG
LncAL713998.1-shRNA-1R	AATTCAAAAAGCTCTGTAACCTTGTGATACTCGAGTATCACAAGGTTACAGAGC
LncAL713998.1-shRNA-2F	CCGGGTTCAAGTTGTCTCTTCAACTCGAGTTGAAGAGACAACTTGAACTTTTTG
LncAL713998.1-shRNA-2R	AATTCAAAAAGTTCAAGTTGTCTCTTCAACTCGAGTTGAAGAGACAACTTGAAC
LncAL138831.2-shRNA-1F	CCGGGATGTTTGCTTTAATTGTACTCGAGTACAATTAAAGCAAACATCTTTTTG
LncAL138831.2-shRNA-1R	AATTCAAAAAGATGTTTGCTTTAATTGTACTCGAGTACAATTAAAGCAAACATC
LncAL138831.2-shRNA-2F	CCGGGGAAGAGCAGCAAGACTGACTCGAGTCAGTCTTGCTGCTCTTCCTTTTTG
LncAL138831.2-shRNA-2R	AATTCAAAAAGGAAGAGCAGCAAGACTGACTCGAGTCAGTCTTGCTGCTCTTCC

Table 2. Primers for qPCR analysis.

Gene	Sequence
LncAL12820.1-qF	TAGTCAAAGGGCTGTGTTGA
LncAL12820.1-qR	AGACTGGACAACAAGGACTT
lnc01984-201-qF	TACAGACCAGCACCGTCC
lnc01984-201-qR	TCTCTTGCCTGGTGACTTTA
lncAC006262.3-qF	GACGGCATCATCATAGCAAC
lncAC006262.3-qR	GACATGTAGGCTCCCAAGT
Lnc02115-qF	CTGTTTGAAGCACGCAGAGT
Lnc02115-qR	TTTGGGGAGTTGTGGGTCTT
lncAL713998.1-qF	CATGCAAGAGACTTCCCTC
lncAL713998.1-qR	CATTGACCGAACACTGAAGG
lncAL138831.2-qF	TACGCCGATGTTTGCTTTAA
lncAL138831.2-qR	ACTACCTGGGGAATGAAACA
GAPDH-qF	CAAACTGTCTTGCCCCACTT
GAPDH-qR	CAGGCAACACCTTACCAACA
